# Is Dialdehyde Chitosan a Good Substance to Modify Physicochemical Properties of Biopolymeric Materials?

**DOI:** 10.3390/ijms22073391

**Published:** 2021-03-25

**Authors:** Sylwia Grabska-Zielińska, Alina Sionkowska, Ewa Olewnik-Kruszkowska, Katarzyna Reczyńska, Elżbieta Pamuła

**Affiliations:** 1 Department of Physical Chemistry and Physicochemistry of Polymers, Faculty of Chemistry, Nicolaus Copernicus University in Toruń, 87-100 Toruń, Poland; olewnik@umk.pl; 2 Department of Chemistry of Biomaterials and Cosmetics, Faculty of Chemistry, Nicolaus Copernicus University in Toruń, 87-100 Toruń, Poland; as@chem.umk.pl; 3 Department of Biomaterials and Composites, Faculty of Materials Science and Ceramics, AGH University of Science and Technology, 30-059 Kraków, Poland; kmr@agh.edu.pl (K.R.); epamula@agh.edu.pl (E.P.)

**Keywords:** dialdehyde chitosan, dialdehyde starch, scaffolds, cross-linking

## Abstract

The aim of this work was to compare physicochemical properties of three dimensional scaffolds based on silk fibroin, collagen and chitosan blends, cross-linked with dialdehyde starch (DAS) and dialdehyde chitosan (DAC). DAS was commercially available, while DAC was obtained by one-step synthesis. Structure and physicochemical properties of the materials were characterized using Fourier transfer infrared spectroscopy with attenuated total reflectance device (FTIR-ATR), swelling behavior and water content measurements, porosity and density observations, scanning electron microscopy imaging (SEM), mechanical properties evaluation and thermogravimetric analysis. Metabolic activity with AlamarBlue assay and live/dead fluorescence staining were performed to evaluate the cytocompatibility of the obtained materials with MG-63 osteoblast-like cells. The results showed that the properties of the scaffolds based on silk fibroin, collagen and chitosan can be modified by chemical cross-linking with DAS and DAC. It was found that DAS and DAC have different influence on the properties of biopolymeric scaffolds. Materials cross-linked with DAS were characterized by higher swelling ability (~4000% for DAS cross-linked materials; ~2500% for DAC cross-linked materials), they had lower density (Coll/CTS/30SF scaffold cross-linked with DAS: 21.8 ± 2.4 g/cm^3^; cross-linked with DAC: 14.6 ± 0.7 g/cm^3^) and lower mechanical properties (maximum deformation for DAC cross-linked scaffolds was about 69%; for DAS cross-linked scaffolds it was in the range of 12.67 ± 1.51% and 19.83 ± 1.30%) in comparison to materials cross-linked with DAC. Additionally, scaffolds cross-linked with DAS exhibited higher biocompatibility than those cross-linked with DAC. However, the obtained results showed that both types of scaffolds can provide the support required in regenerative medicine and tissue engineering. The scaffolds presented in the present work can be potentially used in bone tissue engineering to facilitate healing of small bone defects.

## 1. Introduction

The cross-linking process is widely used for modification of biopolymeric materials [1,2]. There is a need to use cross-linking methods to modify biopolymeric composites physicochemical properties, because of their poor stability in aqueous media (excessive swelling and dissolution), low mechanical strength and nonregularity of pores [3,4]. It is expected that modified materials will be characterized by improved mechanical properties, stability in aqueous environment, improved degradation resistance and biocompatibility [5].

**Figure 1:**
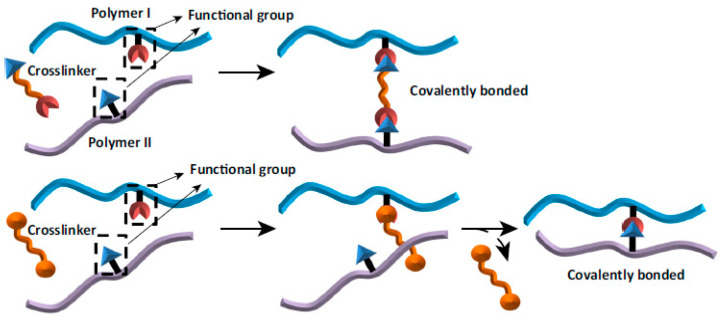
The scheme of chemical cross-linking. Reprinted (with some modifications) from [1] with permission of Elsevier.

There are three cross-linking methods available: chemical, physical and enzymatic [1,2,6]. The most common is chemical modification [5]. It is the process, where covalently bridged polymeric chains are used to improve polymer properties [2,7]. The scheme of chemical cross-linking is shown in Figure 1.

The advantage of chemical cross-linking is formation of very strong bonds between polymeric compounds and the cross-linker. Relative availability of the reagents is also an advantage [2,6]. Chemical cross-linking process is considered to be the most effective and predictable [5]. On the other hand, chemical cross-linking is more expensive than physical modification. The other disadvantage is a need to get rid of the residual cross-linker, because it can be toxic for cells [2,6]. A number of moieties, such as genipin, glutaraldehyde, formaldehyde, dialdehyde starch, dialdehyde alginate, glyoxal, tannic acid, squaric acid, *N*-(3-dimethylaminopropyl)-*N*’-ethylcarbodiimide hydrochloride/*N*-hydroxysuccinimide, i.e., EDC/NHS mixture, can be used as chemical cross-linkers, however their use in biomaterials is limited due to their potential cytotoxicity [1,2,6,8,9,10,11,12,13]. Therefore, there is a need to search for new cross-linking agents that will be used to improve the physicochemical properties of biopolymer materials to be used as biomaterials. These agents have to be safe and nontoxic.

Chitosan (CTS) is a polysaccharide prepared by deacetylation process of chitin. Chitosan–poly[β-(1→4)-2-amino-2-deoxy-D-glucopyranose] is a copolymer of D-glucosamine and N-acetyl-D-glucosamine [14]. Some specific properties of chitosan such as bioactivity, biocompatibility and biodegradability have resulted in increasing interest in its investigation and application in medicine [15,16,17,18,19,20,21]. Derivatives of chitosan have been confirmed to have a unique biological activity and have been used as immunostimulating and antimicrobial agents [14,22,23,24], which are extremely beneficial in the field of biomaterials.

Periodate oxidation of chitosan has gained more attention in recent years. Periodate-oxidized chitosan has been described as a component for achieving biocompatible solid surfaces [25]. The process of periodate oxidation endows chitosan with multiple functional aldehyde groups. Hence, the aldehyde groups might react with the free amino groups within collagen, chitosan and silk fibroin molecules. It was demonstrated for collagen with alginate dialdehyde [26] and collagen with dialdehyde chitosan [27]. The formation of cross-linking bonds between aldehyde groups and amine groups of proteins has been demonstrated for dialdehyde starch [5]. Thus, we hypothesize that it would be feasible to apply dialdehyde chitosan as a novel chemical cross-linker in the modification of biopolymers made of silk fibroin, collagen and chitosan. The dialdehyde chitosan (DAC) is an effective cross-linking agent of collagen materials and cotton fabrics [27,28]. It is expected that the dialdehyde chitosan will be also suitable to cross-link materials containing also silk fibroin and chitosan.

The aim of this work was to obtain materials based on the silk fibroin, collagen and chitosan and cross-link them with dialdehyde starch and dialdehyde chitosan. We presume that obtained materials will have satisfactory mechanical parameters and provide suitable microenvironment for tissue regeneration. Three-dimensional scaffolds based on silk fibroin, collagen and chitosan mixtures, cross-linked with dialdehyde chitosan will be a novel type of biomaterial. It is expected that these materials will be characterized by high mechanical strength, high stability in aqueous conditions and high porosity. These properties guarantee perfect material for the use in tissue engineering.

The materials will be based on readily available and cheap polymers, which can be obtained from food waste, textile waste, and living organisms. The cost of preparation of materials can be relatively low, because the method of obtaining materials (lyophilization) does not require a complicated apparatus. Obtaining the finished material for cell culture will take up relatively short time. An important aspect of the research is the use of waste from the food and textile industries.

## 2. Results and Discussion

### 2.1. Characterization of Dialdehyde Chitosan

The dialdehyde chitosan (DAC) was obtained as a powder soluble in water. In this work, during preparation of dialdehyde chitosan, the weight ratio of oxidant to chitosan was 1:1. It was selected because Węgrzynowska-Drzymalska et al. [29] reported that the highest amount of aldehyde groups was observed in the 1:1 ratio of reagents in the oxidation process. Four different amounts of sodium periodate (weight ratio of oxidant/chitosan = 0.5; 0.7; 0.9; 1.0) were studied and the last one was found to be the best. The results of aldehyde groups content (ALD) determination are shown in Table 1. The average content of aldehyde groups is 57.4 ± 0.85%, which is consistent with Węgrzynowska-Drzymalska et al. [29], where the ALD was 58% and with Keshk et al. [30] where the ALD was 58.8%.

**Figure 2:**
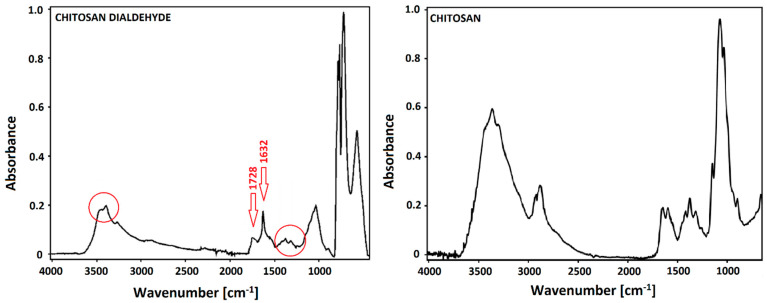
The Fourier transfer infrared spectroscopy with attenuated total reflectance device (FTIR-ATR) spectra of dialdehyde chitosan and chitosan.

The Fourier transfer infrared spectroscopy with attenuated total reflectance device (FTIR-ATR) spectra of dialdehyde chitosan (DAC) and chitosan (CTS) are presented in Figure 2. The band at 1728 cm^−1^ in the left part of Figure 2, is characteristic for the aldehyde groups (carbonyl stretching vibrations) which resulted from the periodate mediated oxidation [31]. The intensity of this peak is closely related to the oxidant/chitosan ratio [31]. At 1632 cm^−1^, a newly formed sharp band can be seen. It derived from carbonyl group and it is a confirmation of chitosan oxidation [29]. By comparison of CTS and DAC spectra, it can be seen that intensity of a peak at 3364 cm^−1^ (Figure 2), which is responsible for O-H stretching vibrations, is lower for dialdehyde chitosan than for chitosan. It is because the cycling structure of chitosan is opened [27,29]. The intensity of the peaks in the range of 1300–1400 cm^−1^ (Figure 2) is also lower for dialdehyde chitosan than for chitosan. It shows effective changes of glucoside rings caused by their opening and oxidation. All results are coherent with literature findings [27,29,31,32].

**Figure 3:**
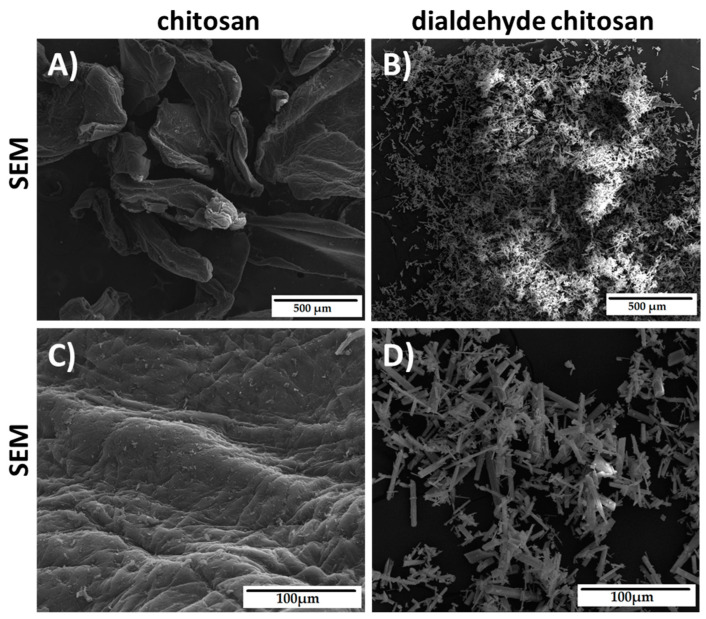
SEM images of chitosan (**A**,**C**) and dialdehyde chitosan (**B**,**D**).

The morphology of chitosan and dialdehyde chitosan was observed by scanning electron microscopy (SEM). The SEM images are shown in Figure 3. As it can be seen, the morphology of CTS and DAC is completely different. The chitosan powder was irregular in shape with rounded edges. After oxidation, chitosan was transformed into structures with pointed needles.

All these observations and conclusions are consistent with the literature reports and prove that dialdehyde chitosan was prepared successfully [27,29,30,31,32].

### 2.2. FTIR-ATR Scaffold Characterization

**Figure 4:**
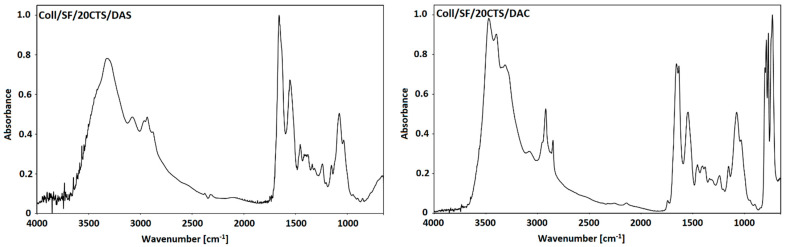
FTIR-ATR spectra of Coll/SF/20CTS scaffold cross-linked by dialdehyde starch (Coll/SF/20CTS/DAS) and dialdehyde chitosan (Coll/SF/20CTS/DAC).

In this research two types of cross-linked materials based on chitosan, collagen, silk fibroin were obtained. First type was cross-linked with dialdehyde starch, while the second type was cross-linked with dialdehyde chitosan. The FTIR-ATR spectra were recorded for both types of scaffolds, i.e., cross-linked by dialdehyde starch and dialdehyde chitosan. Two examples of FTIR-ATR spectra (Coll/SF/20CTS cross-linked with dialdehyde starch and with dialdehyde chitosan) are shown in Figure 4. The positions of characteristic bands for each studied biopolymeric scaffolds are displayed in Table 2.

**Figure 5:**
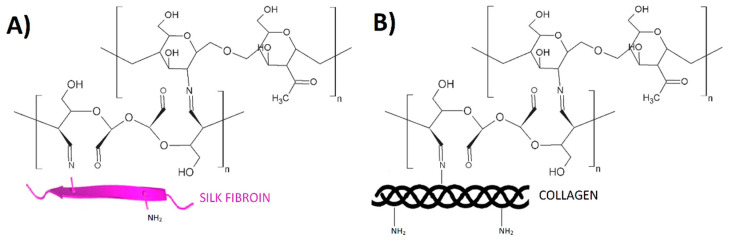
The possible mechanisms of cross-linking between chitosan, a compound with a dialdehyde group (e.g., DAS, DAC) and (**A**) silk fibroin or (**B**) collagen.

The FTIR-ATR analysis was carried out to detect the formation of characteristic bonds resulting from cross-linking reactions. It was previously discussed that the NH– stretching vibration belongs to the amide A bond, near to 3300 cm^−1^ [5]. The frequency of vibrations depends on the strength of hydrogen bonds that stabilize the structure. In the prevailing number of articles the amide I, II and III bands were noticed around 1650 cm^−1^, 1550 cm^−1^, and 1241 cm^−1^, respectively [12]. At ~1075 cm^−1^, C–O stretching vibrations peak provides information about hydrogen bond formation [33]. According to literature [34,35,36], the characteristic absorption peak of imine group, which is the confirmation of cross-linking reaction is present near 1657 cm^−1^. The Schiff’s base formation occurs between the available aldehyde groups of dialdehyde compound and free amino groups of biopolymer [33,34]. Taking into account different blends based on silk fibroin, collagen and chitosan, there are no significant differences in band locations, but some intensity changes of the bands can be observed. Based on the literature [33,37] the possible mechanisms of cross-linking were proposed, which is shown in Figure 5.

### 2.3. Swelling Behavior in Phosphate-Buffered Saline (PBS) and Water Content

**Figure 6:**
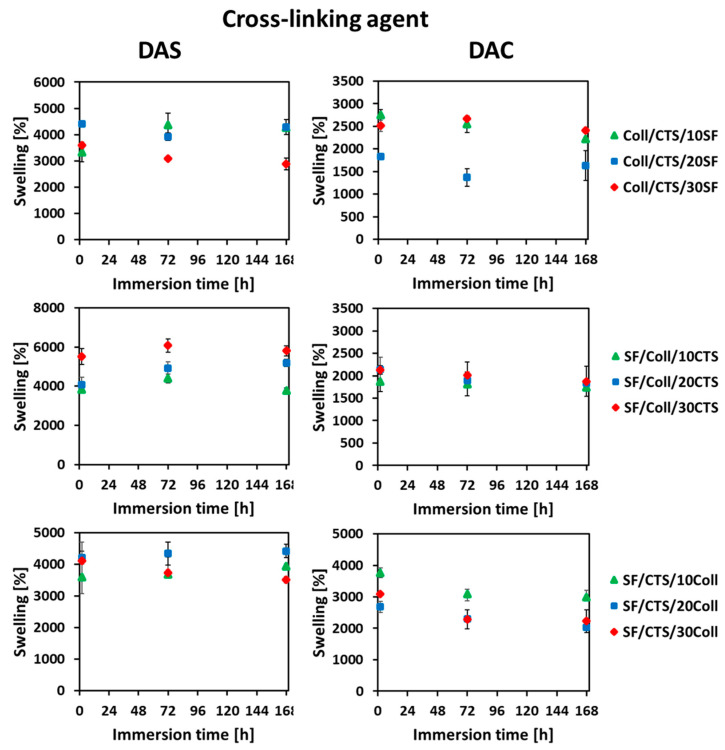
The results of swelling ratio determination of the scaffolds cross-linked with dialdehyde starch (DAS, left side) and dialdehyde chitosan (DAC, right side). Results were presented as mean ± standard error of the mean (*n* = 3).

With the aim to mimic the behavior of the scaffold after its implantation into the human body, the swelling ratio was measured. Based on the obtained results, the swelling ratio curves for the studied materials are shown in Figure 6. The measurements were prepared at three several timepoints: 2 h, 72 h (three days), 168 h (seven days). According to literature [38], the materials based on biopolymers (e.g., silk fibroin, collagen, chitosan, hyaluronic acid) are easily wettable by polar solvents such as PBS or simulated body fluid (SBF) due to presence of functional groups capable of binding water [37,38]. The result is the high swelling degree [38]. There are no previously published reports, where three dimensional materials cross-linked by dialdehyde starch and dialdehyde chitosan were compared.

For each tested scaffold, the swelling ratio increased strongly after 2 h, which is compatible with the previous report [9], where silk fibroin, collagen, 50/50 SF/Coll and 50/50 SF/Coll with 25% addition of chitosan materials were tested. The SF/Coll/25CTS scaffolds cross-linked by dialdehyde starch from our previous article [9] were characterized by high swelling ratio after 1 h (2102 ± 9%). Kuchaiyphum et al. [39] studied silk fibroin/poly(vinyl alcohol) hydrogels cross-linked with dialdehyde starch and they also reported fast growth of swelling ratio in the initial stage of immersion in aqueous media. The collagen, chitosan and silk fibroin have high number of functional groups, which are responsible for wetting capacity by polar solvents. After 2 h of scaffolds immersion in PBS, the swelling ratio was stabilized and it was ~4000% for dialdehyde starch and ~2500% for dialdehyde chitosan. The materials made of collagen and elastin, cross-linked by dialdehyde starch, according to Skopińska-Wiśniewska et al. [5], exhibit stabilized swelling after 24 h. Stabilization of the swelling ratio was also observed for the materials of the earlier cited report of Kuchaiyphum et al. [39], where hydrogels made of collagen and poly(vinyl alcohol) (PVA) mixtures, cross-linked by dialdehyde starch were tested. Węgrzynowska-Drzymalska et al. [29] studied and compared thin films based on chitosan cross-linked by dialdehyde starch, dialdehyde chitosan and glutaraldehyde. They obtained similar results in both with dialdehyde starch and with dialdehyde chitosan cross-linked materials. The swelling ratio increased in the initial stage and stabilized after 6 h of immersion in PBS [29].

Comparing the materials cross-linked with dialdehyde starch and dialdehyde chitosan, it can be concluded that the higher degree of swelling provides cross-linking with dialdehyde starch. However, it should be stressed that in both cases, i.e., DAS and DAC cross-linking, the materials first swelling degree increases rapidly, and then their swelling ability stabilizes.

**Figure 7:**
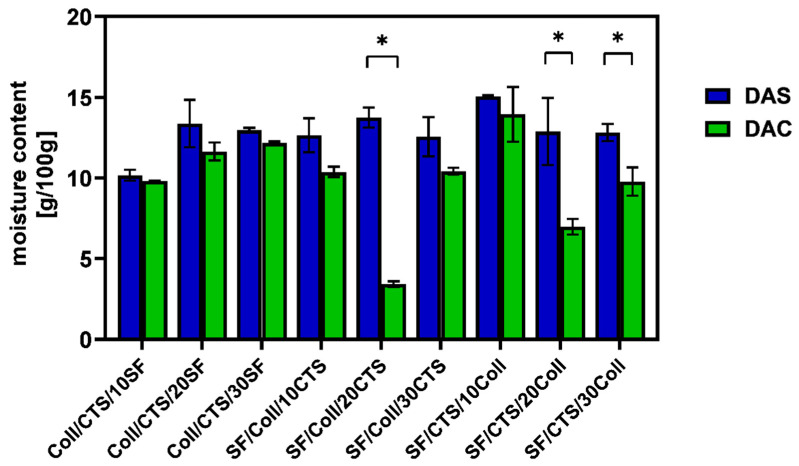
The results of moisture content of the scaffold cross-linked with dialdehyde starch (DAS) and dialdehyde chitosan (DAC). Results are presented as mean ± standard error of the mean (* *p* ≤ 0.05 between cross-linkers, according to one-way ANOVA with Holm–Sidak post hoc test, GraphPad Prism 8.0.1.244).

Water content measurements were used to determine the amount of moisture in the scaffolds. The results of water content determination were expressed as grams of water per 100 g of dry sample and they are shown in Figure 7.

As it can be seen, the materials cross-linked with dialdehyde starch were characterized by a higher amount of water than the materials cross-linked with dialdehyde chitosan. The above-mentioned dependence was observed in each type of studied materials. Generally all materials have been characterized by water content in the range of 9.78 ± 0.88 to 15.06 ± 0.06 g per 100 g dry sample, except SF/Coll/20CTS (3.43 ± 0.17 g/100 g dry sample) and SF/CTS/20Coll (6.98 ± 0.48 g/100 g dry sample) scaffolds cross-linked with dialdehyde chitosan. These results are consistent with Ahmed and Ikram [40], who reported that chitosan biodegradable films were characterized by 16.66% water content. The materials based on biopolymers are characterized by higher moisture content than materials based on other substances, due to availability of free –OH and –NH_2_ groups to interact with water [40,41]. Revati et al. [42] found that porous scaffolds made of biodegradable polylactic acid with addition of *Pennisetum purpureum* as a filler, had moisture content in the range of 0.5 to 1.2%, depending on the scaffold composition. If it is about hydrogels, which are characterized by high water content compared to scaffolds obtained by lyophilization, Zhou et al. [11] reported moisture content about 70% for materials based on poly(vinyl alcohol)/silk fibroin/nano-hydroxyapatite cross-linked with genipin. Comparing results from this study to the results from our previous report, where three-component silk fibroin, collagen, chitosan based materials were cross-linked by glyoxal solution, it can be seen that observations are consistent [43]. Referring to our previous article, when we checked the impact of cross-linking agent to physicochemical properties of silk fibroin/collagen and silk/fibroin/collagen with 25% addition of chitosan materials, it can be concluded that cross-linking agents reduce water content in porous scaffolds [9].

To sum up, materials based on silk fibroin, collagen and chitosan mixtures, cross-linked with dialdehyde starch were characterized by higher amount of water than materials cross-linked with dialdehyde chitosan.

### 2.4. Porosity and Density

In order to evaluate whether the obtained materials are suitable for tissue engineering purposes, their porosity and density were measured. The obtained results are shown in Table 3.

According to Kaczmarek et al. [44], the appropriate porosity to use material in bone tissue engineering, is about 80–90%. This porosity value may improve the cells and nutrition flow though the scaffold which enhance tissue regeneration [45]. All scaffolds tested in this experiment meet this criterion. The highest porosity, was noticed to SF/Coll/10CTS scaffold cross-linked with dialdehyde chitosan (94.2 ± 1.3%), while the lowest porosity to SF/CTS/20Coll scaffold cross-linked with dialdehyde starch (83.8 ± 1.0%). To compare dialdehyde starch and dialdehyde chitosan influence on porosity, there is no significant difference between the results obtained for materials cross-linked by both dialdehydes. Kaczmarek et al. [46] studied scaffolds based on chitosan, collagen and hyaluronic acid cross-linked with dialdehyde starch, and they reported that dialdehyde starch reacts with the polymeric chains. It resulted in structural changes, which improved the total material porosity [46]. Yuan et al. [47] have studied gelatin-based scaffolds cross-linked by genipin with presence of dialdehyde amylose, and they reported porosity in the range of 87.46–92.53%, which is consistent with our results. Porosity of 95.14% was observed for dialdehyde cellulose nanocrystal/gelatin hydrogel, following to Jiang et al. [48]. The porosity is comparable with our previous results on materials based on silk fibroin, collagen and chitosan cross-linked with glyoxal solution [43].

The density of scaffolds cross-linked with dialdehyde chitosan is higher than for scaffolds cross-linked with dialdehyde starch, excluding materials based on 50/50 SF/CTS mixture with addition of collagen. Kaczmarek et al. [46] reported density of chitosan/collagen/hyaluronic acid scaffolds (without and with presence of hydroxyapatite) in the range of 6.14 ± 0.11 to 8.42 ± 0.21 g/mL, which is much lower than reported in this paper. The highest density was observed for SF/CTS/30Coll scaffold cross-linked with dialdehyde chitosan 21.8 ± 2.4 g/mL), while the lowest for SF/Coll/30CTS scaffold cross-linked with dialdehyde chitosan (11.1 ± 0.3 g/mL). The density for two- and three-component materials is higher than for one component collagen-based materials, even if it was cross-linked with dialdehydes, which can be seen in our previous study, where collagen scaffolds cross-linked by dialdehyde starch with magnetic particles addition was studied [49].

### 2.5. Scanning Electron Microscopy

**Figure 8:**
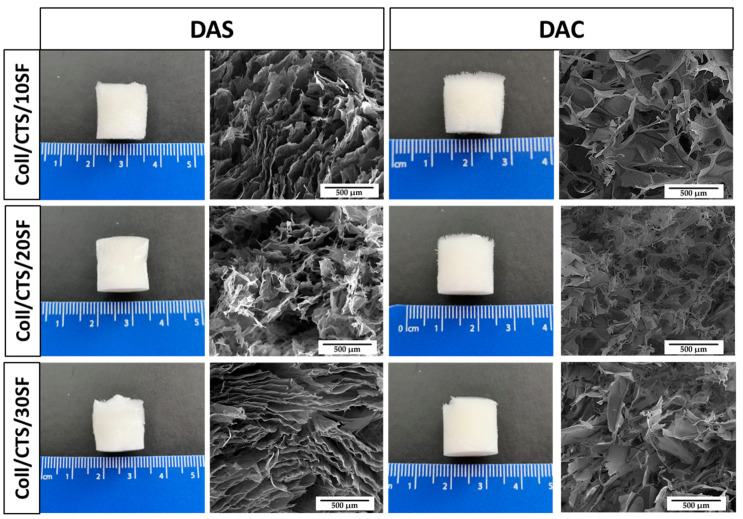
Macroscopic appearance and SEM micrographs of 50/50 Coll/CTS scaffolds with 10, 20 and 30% addition of silk fibroin cross-linked with dialdehyde starch (DAS) and dialdehyde chitosan (DAC).

**Figure 9:**
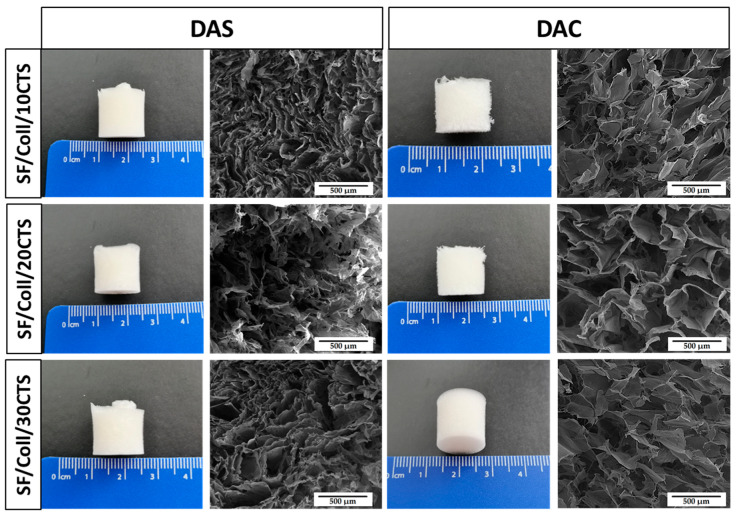
Macroscopic appearance and SEM micrographs of 50/50 SF/Coll scaffolds with 10, 20 and 30% addition of chitosan cross-linked with dialdehyde starch (DAS) and dialdehyde chitosan (DAC).

**Figure 10:**
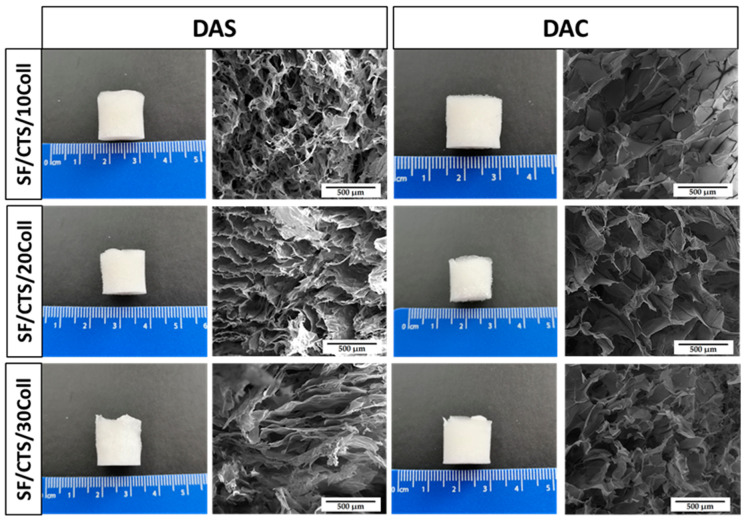
Macroscopic appearance and SEM micrographs of 50/50 SF/CTS scaffolds with 10, 20 and 30% addition of collagen cross-linked with dialdehyde starch (DAS) and dialdehyde chitosan (DAC).

In an aim to observe microstructure of cross-linked biopolymeric scaffolds, scanning electron microscopy imaging was performed. The images are shown in Figure 8, Figure 9 and Figure 10. The magnification of ×150 was used in this study. Macroscopic images of the scaffolds were taken and are shown in Figure 8, Figure 9 and Figure 10.

As it can be seen in SEM micrographs (Figure 8, Figure 9 and Figure 10), each type of scaffold has a porous structure. The pores were interconnected. The materials were looking similar to previously exanimated scaffolds fabricated from silk fibroin/collagen/hyaluronic acid [50], silk fibroin/gelatin/chitosan [51], chitosan/poly(glycerol sebacate) [52] or silk fibroin/sodium alginate [53]. The porous structure with interconnected pores is suitable for scaffolds for biomedical applications, especially in bone tissue regeneration. It is very important, because they facilitate the transport of gases, nutrients and components secreted by the cells [54]. It can be concluded that there are no significant differences in the macroscopic appearance of the scaffolds as all of them look similar (white cylindrical, regular shape). Nevertheless, the differences are visible in the SEM micrographs. Thanks to the program provided by the SEM manufacturer, the pore size could be approximately analyzed [55]. The scaffolds cross-linked with dialdehyde chitosan, were characterized by bigger pores than scaffolds cross-linked with dialdehyde starch.

### 2.6. Mechanical Properties

**Figure 11:**
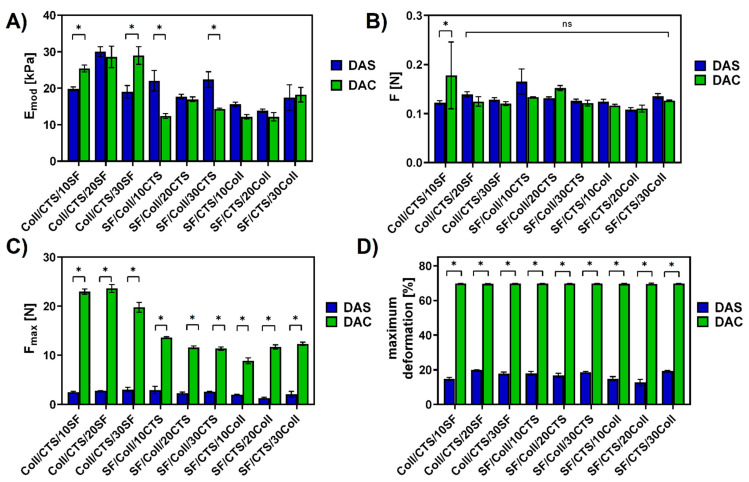
Mechanical parameters of the scaffolds cross-linked with dialdehyde starch (DAS) and dialdehyde chitosan (DAC): (**A**) Young’s modulus (E_mod_); (**B**) force at maximum deformation (F); (**C**) maximum force (F_max_); (**D**) maximum deformation. Results are presented as mean ± standard error of the mean (* *p* ≤ 0.05 between cross-linkers; ns: no significant difference according to one-way ANOVA with Holm–Sidak post hoc test, GraphPad Prism 8.0.1.244).

Mechanical properties of the matrix offer essential support to cells and play a key role in the cells’ growth and differentiation, but this is only a transition stage of the material [56]. In order to observe the differences between dialdehyde starch and dialdehyde chitosan cross-linking materials, the mechanical resistance at room conditions (temperature, humidity) were tested. The mechanical parameters Young’s modulus (E_mod_), force at maximum deformation (F), maximum force (F_max_) and maximum deformation were determined and compared. The results are presented in Figure 11.

As it can be seen in Figure 11, scaffolds cross-linked with dialdehyde starch had higher Young’s modulus than scaffolds cross-linked with dialdehyde chitosan. This observation is in agreement with Wegrzynowska-Drzymalska et al. [29], where chitosan films were cross-linked with dialdehyde starch, dialdehyde chitosan and glutaraldehyde. They reported higher Young’s modulus for chitosan cross-linked with 10% of dialdehyde starch than with 10% of dialdehyde chitosan. Only for materials based on 50/50 Coll/CTS with addition of silk fibroin, Young’s modulus was comparable (Coll/CTS/20SF) or higher (Coll/CTS/10SF, Coll/CTS/30SF) for dialdehyde chitosan cross-linking. Taking into consideration force at maximum deformation, there were no significant differences between majority of the studied materials (Figure 11B). Only for scaffolds fabricated from 50/50 Coll/CTS mixture with 10% addition of silk fibroin showed a difference, and it was higher for material cross-linked with dialdehyde chitosan. The maximum force was significantly higher for composites cross-linked with dialdehyde chitosan than cross-linked with dialdehyde starch (Figure 11C). The same trend was observed in the case of maximum deformation (Figure 11D). Materials cross-linked with dialdehyde chitosan were maximally deformed in ~69%, while materials cross-linked with dialdehyde starch in the range of 12.67 ± 1.51% to 19.83 ± 1.30%. Following to Asadpour et al. [51], where mechanical properties of silk fibroin/chitosan/gelatin scaffolds in wet conditions were studied, the tensile strength (from 7.4 to 18 kPa) and elastic modulus (from 27 to 60 kPa) gradually increased when the silk fibroin content was increased. According to Singh and Pramanik [57] the chitosan-based scaffolds with addition of silk fibroin fibers (1.02  ±  0.04 MPa and 1.76  ±  0.11 MPa) possess superior compressive strength in comparison to pure chitosan scaffold (0.45  ±  0.04 MPa). In this report, they observed that the compressive strength of scaffold increases as the silk fibroin fibers content increases, which is in agreement with our results for 50/50 Coll/CTS with addition of silk fibroin materials [57]. Taking into account, materials based on silk fibroin and chitosan, prepared by Hu et al. [58], the chitosan/silk fibroin composites with hydroxyapatite addition, displayed better mechanical properties with the increasing amount of silk fibroin.

### 2.7. Thermal Properties

In an aim to study the stability after high temperature treatment, thermogravimetric analysis of scaffolds were performed in the range from 20 °C to 600 °C [59,60,61]. The results can be seen in Table 4.

Table 4 clearly showed that the materials cross-linked by means of dialdehyde chitosan were more stable than materials cross-linked with dialdehyde starch. The residue after heating, was higher for materials cross-linked with dialdehyde chitosan for each type of the scaffold. The weight loss during heating process occurred due to water evaporation, detachment of side hydroxyl groups, main chain scission, and pyranose ring-opening reactions [29]. Intensive destruction of the structure has occurred. The highest weight loss after high temperature treatment was observed to SF/Coll/10CTS scaffold cross-linked with dialdehyde starch (25.22% residue), while the lowest to was found to Coll/CTS/20SF scaffold cross-linked with dialdehyde chitosan (41.82% residue). Sharmila et al. [62] reported 22.2% and 27% of residue for alginate/carboxymethyl cellulose scaffolds (with addition of *Spinacia oleracea* extract and *Cissus quadrangularis* extract) for bone tissue engineering. Our results of residue after heating, can be compared with the collagen residue from León-Mancilla et al.’s article [63]. They also performed thermogravimetric analysis of bone matrix, where 63.59% residue was noticed [63]. Comparable results of residue measurements were obtained by Zhang et al. [64] for materials of silk fibroin/hydroxybuthyl chitosan blended nanofibers (in the range of 17.99 to 26.42%).

### 2.8. Cytotoxicity and Cell Attachment

**Figure 12:**
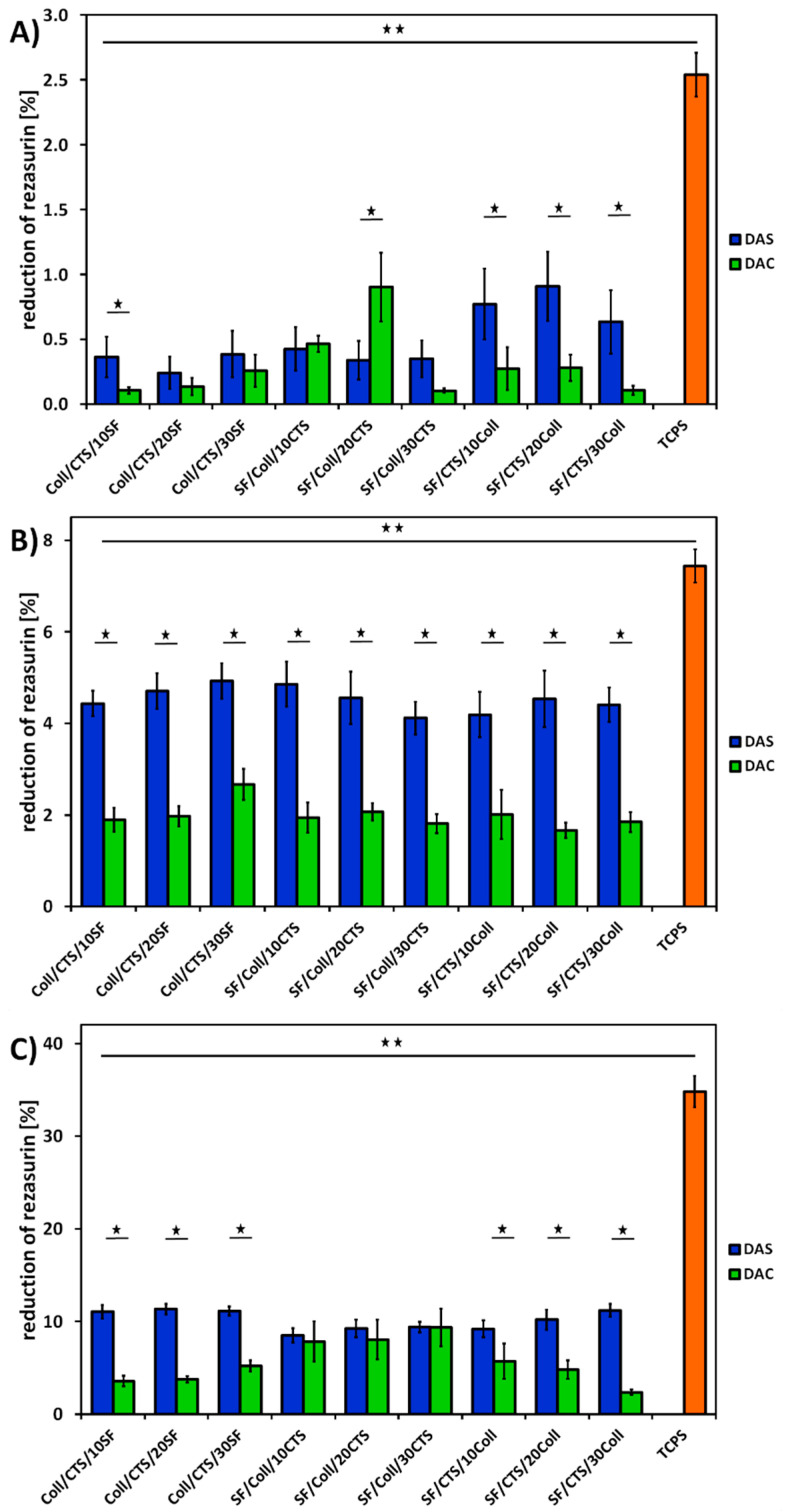
Metabolic activity expressed as reduction of AlamarBlue on days 1 (**A**), 3 (**B**), 7 (**C**) of MG-63 cells cultured on scaffolds cross-liked with dialdehyde starch (DAS) and dialdehyde chitosan (DAC) and on control TCPS (tissue culture polystyrene). Results are presented as mean ± standard error of the mean (* *p* ≤ 0.05 between cross-linkers; ** *p* ≤ 0.05 vs. TCPS according to one-way ANOVA with Holm–Sidak post hoc test, GraphPad Prism 8.0.1.244).

In order to evaluate cytocompatibility of three-component biopolymeric scaffolds cross-linked with dialdehyde starch and dialdehyde chitosan, metabolic activity assay with MG63 cells was performed. The results of AlamarBlue reduction assay are shown in Figure 12.

Based on Figure 12, it can be seen that the cells cultured on tissue culture polystyrene (TCPS) showed the highest reduction of AlamarBlue. It is because TCPS is regarded as an ideal substrate for cell adhesion and proliferation. In the case of TCPS, all of the cells initially seeded remained in the well. If it is about scaffolds, the cells were seeded directly on the scaffolds, but some of them adhered to the bottom of the well, because the scaffolds were slightly smaller than the well itself. The activity of the cells cultured on TCPS was measured throughout the experiment, while the scaffolds were transferred into a new plate prior to testing cell metabolic activity.

As it can be seen in Figure 12, better results were obtained for materials cross-linked with dialdehyde starch. After the first day of cell culture (Figure 12A), materials of each type, except SF/Coll/10CTS and SF/Coll/20CTS, were characterized by higher resazurin reduction for dialdehyde starch than for dialdehyde chitosan cross-linking. Looking at the third day of cell culture (Figure 12B), it can be seen better cell response for each scaffolds cross-linked with dialdehyde starch than for the scaffolds cross-linked with dialdehyde chitosan. The level of resazurin reduction is approximately two times higher for materials cross-linked with DAS than with DAC. Taking into account seven days of cell culture (Figure 12C), some differences between all types of materials could be noticed. For materials based on 50/50 SF/Coll mixture with addition of CTS, comparable results were observed. Similar cell response was observed for these materials cross-linked with two various cross-linking agents. On the other hand, for the rest of materials, similar conclusion to observations after first and third day of cell culture can be drawn. Better cell responses were noticed for dialdehyde starch cross-liked materials (twice and sometimes even three times). Therefore, there is no toxic effect of the cross-linking agent on cell viability, an increase in resazurin reduction is visible in the following days of cell culture.

**Figure 13:**
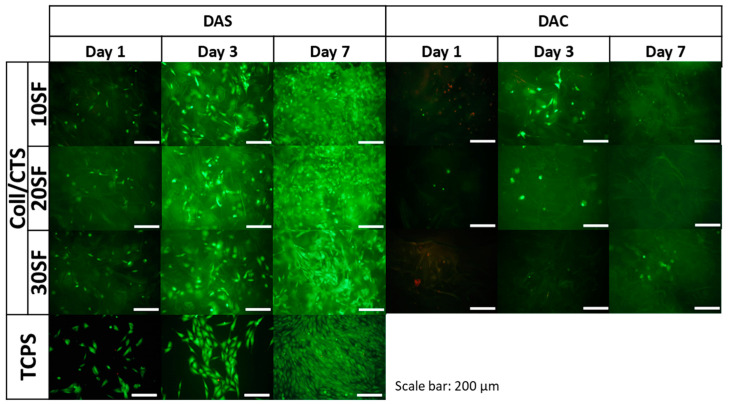
Images of MG-63 cells grown for one, three or seven days on Coll/CTS 50/50 mixtures with 10, 20 and 30% addition of SF, cross-linked with dialdehyde starch (DAS) and dialdehyde chitosan (DAC) scaffolds and on control TCPS. Live/dead staining. Scale bar = 200 μm.

**Figure 14:**
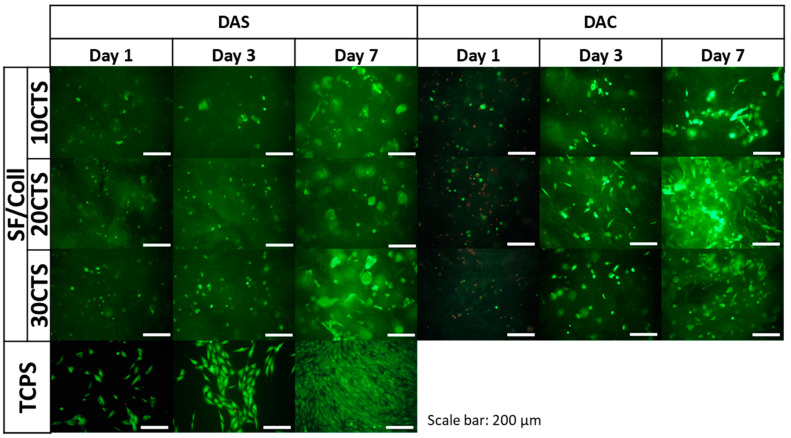
Images of MG-63 cells grown for one, three or seven days on SF/Coll 50/50 mixtures with 10, 20 and 30% addition of CTS, cross-linked with dialdehyde starch (DAS) and dialdehyde chitosan (DAC) scaffolds and on control TCPS. Live/dead staining. Scale bar = 200 μm.

**Figure 15:**
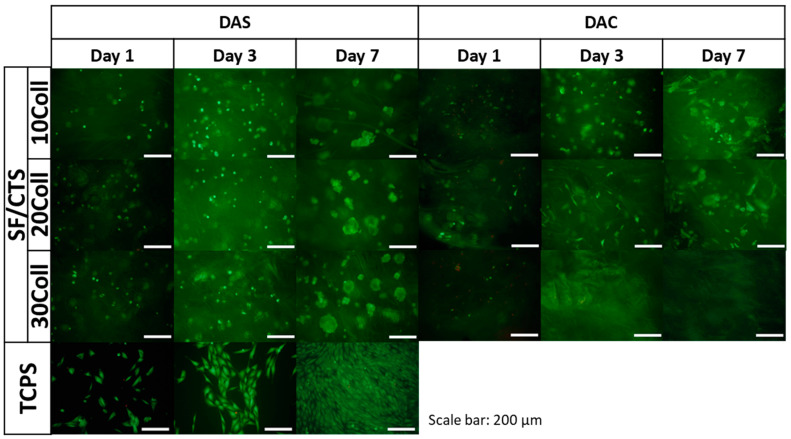
Images of MG-63 cells grown for one, three or seven days on SF/CTS 50/50 mixtures with 10, 20 and 30% addition of Coll, cross-linked with dialdehyde starch (DAS) and dialdehyde chitosan (DAC) scaffolds and on control TCPS. Live/dead staining. Scale bar = 200 μm.

The results of metabolic activity were further confirmed by a live/dead fluorescent staining and its results are presented in Figure 13, Figure 14 and Figure 15. The figures show the morphology of live (green) and dead (red) cells.

As it can be seen, the number of cells visible on the samples on day 1 was low, but, as the cell culture time increased, the number of cells gradually increased. Live cells can be visible on the scaffolds at all time points. When it comes to comparison of cross-linking agents, the samples cross-linked with dialdehyde starch were characterized by higher number of cells at all timepoints, which is consistent with metabolic activity assay. However, even if dialdehyde chitosan cross-linked samples are considered, the live cells are visible in each type of material. It can be also observed that some cells migrated into deeper parts of the three-dimensional material.

For Coll/CTS 50/50 with addition of silk fibroin, cross-linked with dialdehyde starch, morphology of cells was similar to the cells cultured on control TCPS and they were characterized by spindle, elongated shape. The same materials cross-linked with dialdehyde chitosan did not show such a good cytocompatibility and did not support cell attachment and proliferation. However, in the case of SF/Coll samples with the addition of different amounts of chitosan, the negative influence of cross-linking agents was not observed, as the number of cells observed on the surface of both DAS and DAC cross-linked hydrogels was similar. What is more, the cells growing on DAC modified samples exhibited more spindlelike morphology, while the majority of the cells cultured on DAS cross-linked sponges were clustered in agglomerates. As regards SF/CTS samples with different concentration of collagen, fewer cells were found on DAC modified hydrogels. Nonetheless, in all cases the number of cells increased in the course of time and no significant numbers of dead cells were found, except from Coll/CTS/SF cross-linked with DAC.

Kaczmarek et al. [33] reported that addition of dialdehyde starch to scaffolds fabricated based on chitosan and gelatin mixture did not show effect to SaOs-2 cells and they attached and proliferated normally on scaffolds. According to Parekh et al. [65] no significant differences between silk fibroin/collagen and silk fibroin/chitosan materials in viability of the cells were observed. Rajalekshmi et al. [66] studied fibrin incorporated alginate dialdehyde-gelatin hydrogels and they reported cytocompatible nature of hydrogels with mouse fibroblast cells (L929). In turn, Muchova et al. [67] used mouse embryonic fibroblast cell line (NIH/3T3) to evaluate dialdehyde cellulose influence to poly(vinyl alcohol) hydrogels. They reported that dialdehyde cellulose did not have any observable impact on the cytotoxicity of hydrogels and all tested materials were nontoxic after 48 and 96 h of cell culture [67]. Following to Chen et al. [68], membranes based on collagen, cross-linked with oxidized chitosan oligosaccharide, have a good biocompatibility. They evaluated toxicity of the materials by MTT (3-(4,5-dimethylthiazol-2-yl)-2,5-diphenyl-2H-tetrazolium bromide) assay with L929 fibroblast cells. It was noticed that higher amount than 2% of cross-linking agent, increased cytotoxicity effect of the material, however thanks to confocal microscopy they observed a little adverse effect on cell growth and proper proliferation of the cells was visible [68]. The fibroblast cells grow well with proper morphology on the surface of the membranes cross-linked with oxidized chitosan oligosaccharide [68].

To sum up, none of the dialdehyde starch cross-linked tested samples showed toxicity effects on MG63 cells. The number of cells growing on the scaffold increased with time. The cells grown on the examined scaffolds proliferated properly. Considering the materials cross-linked with dialdehyde chitosan, it should be noted that they were in a weaker environment for cell growth. After first day of cell culture, the number of cells decreased, but it did not affect to their metabolic processes, the number of cells increased after three and seven days.

As it is known, some compounds containing aldehyde groups are harmful for cells, due to their possible toxicity and leakage of unreacted cross-linker molecules in vivo (formaldehyde, glutaraldehyde, or glyceraldehyde can be an examples [9,68]). Thus, novel and safer cross-linking agents, obtained from natural organic compounds with dialdehyde groups are extensively investigated. Unlike that of formaldehyde, glutaraldehyde and glyceraldehyde, according to Chen et al. [68] and Hu et al. [26], studied dialdehydes (oxidized chitosan oligosaccharide, dialdehyde chitosan, dialdehyde starch, dialdehyde cellulose, dialdehyde alginate) have a skeleton structure, which could avoid the harmful influence of aldehyde groups.

The main limitation of this experiment was the appropriate preparation of dialdehyde chitosan as a cross-linking agent. It was because chitosan dialdehyde was prepared in our laboratory, unlike dialdehyde starch which was bought from Sigma-Aldrich. Moreover, it was very important to check the cellular response to the used cross-linking agents. Since one of the major disadvantages of chemical cross-linking might be unreacted residues of cross-linker remaining in the material.

## 3. Materials and Methods

Reagents purchased form Sigma-Aldrich (St. Louis, MO, USA): chitosan (DD = 78%; viscosity average molecular weight = 0.59 × 10^6^ g/mol), acetic acid, hydrochloric acid, sodium carbonate, lithium bromide, calcium chloride dihydrate, acetone, sodium periodate, sodium hydroxide, phosphate buffer saline (PBS), purchased in a tablet form with one tablet dissolved in 200 mL of deionized water yields 0.01M phosphate buffer, 0.0027 M potassium chloride and 0.137 M sodium chloride, pH = 7.4, at 25 °C).

Dialdehyde starch (potato origin, Mv = 188 g/mol, ALD = 67%) was purchased from Chemos GmbH&Co. KG (Altdorf, Germany).

### 3.1. Fabrication of Dialdehyde Chitosan

The synthesis of dialdehyde chitosan was prepared following Bam et al. [69] with the slight modifications used in Węgrzynowska-Drzymalska et al.’s work [29]. Chitosan was dissolved in 0.1 M acetic acid solution to obtain 1% concentration. Appropriate quantity of 0.3 M aqueous solution of sodium periodate (weight ration of oxidant/chitosan = 1) was added to the chitosan solution under magnetic stirring. After heating the mixture to 40 °C, stirring was continued for 3 h in the dark conditions. After the mixture has cooled down, the appropriate amount of acetone, was poured to obtain precipitate. The precipitate of dialdehyde chitosan was filtered and dried 24 h in room conditions (temperature, humidity).

### 3.2. Fabrication of Scaffolds

Collagen (Coll) was obtain in our laboratory. The protocol was described in detail in our previous paper [70]. Briefly, tail tendons of young rats were separated, washed by distilled water and dissolved in 0.1 M acetic acid. Undissolved parts were removed by centrifuging, proper solution was frozen and lyophilized to obtain 100% collagen. Chitosan (CTS) and collagen were dissolved in 0.1M acetic acid, to obtain 1% solutions. Silk fibroin (SF) was prepared in our laboratory from *Bombyx mori* cocoons. The methodology was described in detail in our previous work [9]. Shortly, the cocoons were boiled in a Na_2_CO_3_ aqueous solution (1 h, 2×), in an alkaline soap solution (30 min) and in distilled water (20 min). Cocoons were rinsed with tap (three times) and distilled (three times) water. The procedure was repeated three times. The degummed silk was dried at room temperature and humidity (48 h). Silk fibroin was dissolved in 9.3 M lithium bromide to obtain 5% solution.

**Figure 16:**
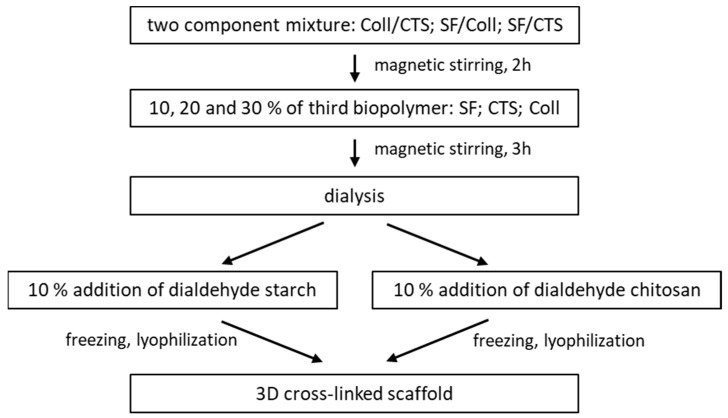
The scaffolds preparation scheme.

The scheme of scaffold preparation is shown in Figure 16. Two biopolymers (Coll/CTS; SF/Coll; SF/CTS) were mixed together in 50/50 weight ratio with a magnetic stirrer (2 h). The third biopolymer (SF; CTS; Coll) was added in 10, 20 and 30 *w*/*w*% amount and mixed (3 h). The mixtures were dialyzed against distilled water (three days) to aqueous solutions. The cross-linking agents: dialdehyde starch, dialdehyde chitosan) were added to obtained mixtures and mixed with a magnetic stirrer (3 h). The mixtures were poured to 24-well plates and frozen (one day, −80 °C) and lyophilized (−55 °C, 5 Pa, 48 h, ALPHA 1–2 LD plus, CHRIST, Osterode am Harz, Germany).

### 3.3. Characterization of Dialdehyde Chitosan

The number of aldehyde groups content (ALD, %) was determined by acid-based titration [71]. Dialdehyde chitosan (0.1 g) and NaOH solution (5 mL, 0.25 M) were poured in an Erlenmeyer flask, and the system was heated to 70 °C for 2 min, until the sample was dissolved. After rapid cooling, HCl solution (7.5 mL, 0.25 M) and distilled water (15 mL) were added. The appropriate amount of phenolphthalein indicator was added and the end point was titrated with NaOH solution (0.25 M). This procedure was repeated three times. The following formula (1) was used to calculate the dialdehyde content:

(1) $$ALD=\frac{C_{1}V_{1}-C_{2}V_{2}}{m/M}\times 100\%\;\left[\%\right]$$

where:

C_1_—the concentration of NaOH solution [mol/dm^3^],
V_1_—the volume of NaOH solution [dm^3^],
C_2_—the concentration of HCl solution [mol/dm^3^],
V_2_—the volume of HCl solution [dm^3^],
m—mass of the sample [g],
M—molecular weight of the repeated unit in dialdehyde chitosan [M = 160g/mol].

The structure of chitosan and synthetized dialdehyde chitosan was evaluated by attenuated total reflection infrared spectroscopy with an attenuated total reflectance (FTIR-ATR) device with germane crystal (Nicolet iS10, Thermo Fisher Scientific, Waltham, MA, USA). Spectra were recorded in 64 scans in absorption mode at 4 cm^−1^, in the range of 400—4000 cm^−1^.

The morphology of the chitosan and prepared cross-linking agent was characterized by scanning electron microscopy (SEM, LEO Electron Microscopy, Ltd., England, UK).

### 3.4. Fourier Transform Infrared Spectroscopy (FTIR)

Nicolet iS10 spectrophotometer equipped with an attenuated total reflectance (FTIR-ATR) device with a germanium crystal (Nicolet iS10, Thermo Fisher Scientific, Waltham, MA, USA) was used to observe the chemical structure of the obtained scaffolds. The spectra were evaluated in the range of 600–4000 cm^−1^. All spectra were recorded with the resolution of 4 cm^−1^ with 64 scans.

### 3.5. Swelling Behavior in Phosphate-Buffered Saline (PBS) and Water Content

The swelling behavior was carried out by immersing the scaffolds fragments in PBS (10 mL, pH = 7.4, 37 °C). After two hours, three days and seven days of immersion, materials were put between two sheets of paper and weighted [49]. The following Equation (2) was used to obtain swelling degree:

(2) $$swelling=\frac{\left(m_{s\left(t\right)}-m_{s\left(0\right)}\right)}{m_{s\left(0\right)}}\times 100\%\;\left[\%\right]$$

where:

m_s(t)_ is the weight of the material after immersion in PBS [g],
m_s(0)_ is the weight of the material before immersion [g].

Water content of scaffolds was measured by drying samples at 105 °C until they reached a constant weight. The results were expressed as grams of water per 100 g of dry sample [43].

Samples of each type were measured in triplicate (*n* = 3).

### 3.6. Porosity and Density

Liquid displacement method with isopropanol was used to measure porosity and density of the scaffolds. A fragment of the sample with a known weight was immersed in a cylinder with a known volume of isopropanol for 3 min. The porosity was calculated using this Equation (3):

(3) $$\varepsilon =\frac{V_{1}-V_{3}}{V_{2}-V_{3}}\times 100\%\;\left[\%\right]$$

where:

V_1_—initial volume of isopropanol [cm^3^],
V_2_—total volume of isopropanol with the isopropanol impregnated sample [cm^3^],
V_3_—volume of isopropanol after scaffold removal [cm^3^].

The density was calculated using following Equation (4):

(4) $$d=\frac{W}{V_{2}-V_{3}}\;\left[\frac{mg}{cm^{3}}\right]$$

where:

W—weight of sample [mg],
V_2_, V_3_—as above.

For each kind of material, three samples were measured.

### 3.7. Scanning Electron Microscopy

Structure of the scaffolds cross-linked with dialdehyde starch and dialdehyde chitosan was analyzed using scanning electron microscope (model 1430 VP, LEO Electron Microscopy Ltd., England, UK). In an aim to obtain high quality images, the samples were cut using liquid nitrogen and their cross-section was covered with a thin layer of gold. Micrographs of all samples were taken at 150× magnification.

### 3.8. Mechanical Properties

The mechanical properties studies of scaffolds were prepared using a Zwick & Roell 0.5 testing machine (Zwick&Roell Group, Ulm, Germany). The studies were performed at crosshead speed = 0.5 mm/min at room temperature and humidity. The scaffold was placed between two discs of the testing machine and compressed. For each kind of material, five samples were measured. The thickness of the samples was in the range of 13–15 mm with the diameter 14.44 mm.

### 3.9. Thermal Properties

Thermogravimetric analysis (TGA) of scaffolds were performed using a Thermal Analysis SDT 2960 Simultaneous TGA-DTA analyzer (TA Instruments, New Castle, De, USA) in the range from 20 °C to 600 °C at a heating rate of 20 °C/min in the atmosphere of nitrogen [59,60,61].

### 3.10. Cell Seeding on Composite Scaffolds

The procedure of biological tests was described in detail in our previous paper [9]. The scaffolds (height = 2–4 mm, diameter = 14.44 mm) were sterilized by soaking in ethanol solution (70%) for 10 min and washed (5×) with sterile PBS (pH = 7.4). Cytocompatibility of the scaffolds was studied using MG-63 osteoblast-like cells (European Collection of Cell Cultures, Salisbury, UK). The cells were cultured in Eagle’s minimal essential medium (EMEM, PAN BIOTECH, Aidenbach, Germany) supplemented with fetal bovine serum (10%), penicillin-streptomycin (1%), sodium pyruvate (0.1%) and nonessential amino acids (0.1%). The cells were cultured in a humidified atmosphere (5% CO_2_) at 37 °C. The scaffolds were seeded at 1 × 10^4^ cells per sample (suspended in 1 mL of EMEM). As a control, the cells seeded directly on tissue culture polystyrene (TCPS, Nunclon) were used. Three repetitions were made for each type of sample.

### 3.11. Metabolic Activity and Cells Attachment

Metabolic activity of the cells was determined using resazurin reduction assay, after one, three and seven days. Cell culture medium was carefully removed from the samples and changed with 1 mL of fresh cell culture medium containing 5% of AlamarBlue (resazurin) reagent (Sigma Aldrich). After incubation (3 h) the medium (100 µL) was transferred into a black 96-well plate for fluorescence measurement (λ_ex_ = 544 and λ_em_ = 590 nm, FluoStar Omega, BMG Labtech, Ortenberg, Germany). The following formula (5) was using to calculation of percentage resazurin reduction:

(5) $$reduction\;of\;resazurin=\frac{S^{x}-S^{control}\;}{S^{100\%reduced}-S^{control}}\times 100\%\;\left[\%\right]$$

where:

S^x^—fluorescence of the samples,
S^control^—fluorescence of EMEM with 5% AlamarBlue reagent, without cells (0% reduction of resazurin),
S^100%reduced^—fluorescence of EMEM with 5% AlamarBlue reagent autoclaved at 121 °C for 15 min (100% reduction of resazurin).

Live/dead staining was carried out to observe cell attachment, spreading and viability. The samples were washed with PBS and stained using calcein AM (0.1%, Sigma Aldrich) and propidium iodide (0.1%, Sigma Aldrich) and incubated for 20 min (37 °C). Axiovert 40 (Zeiss, Oberkochen, Germany) microscope with HXP 120 C Metal Halide Illuminator (Zeiss, Oberkochen, Germany) was used to take fluorescence microscopy images.

## 4. Conclusions

In summary, silk fibroin, collagen, chitosan three-dimensional scaffolds were cross-linked with dialdehyde starch and dialdehyde chitosan and processed by freeze-drying method. The dialdehyde chitosan was prepared by one step synthesis with sodium periodate. The number of dialdehyde groups analysis, FTIR-ATR spectroscopy and SEM images were performed to characterize dialdehyde chitosan. The comparison of physicochemical properties and metabolic activity of SF-, CTS-, and Coll-based scaffolds, cross-linked with dialdehyde starch and dialdehyde chitosan were reported. The resulting properties depend on scaffold composition and cross-linking agent.

It was found that both dialdehyde starch and dialdehyde chitosan have an impact on mechanical properties, pore size, swelling ability and thermal stability of the scaffolds. The majority of materials were cytocompatible with MG-63 cells, however the scaffolds cross-linked with dialdehyde starch provided a better environment to culture of MG-63 cells.

It can be concluded that dialdehyde chitosan can be proposed as a cross-linker to biopolymeric matrices for bone tissue engineering. The cross-linking of three-component biopolymeric mixtures with dialdehyde chitosan may be a new method of modification of biopolymeric scaffolds. Before dialdehyde chitosan will be widely used as modifying agent to improve physicochemical properties of the materials, more research should be performed. Future studies could be focused on the usefulness of chitosan dialdehyde in modification of other biopolymers. More studies can be done to find out what is the limit of this cross-linker used to obtain a “safe” material for application in tissue engineering. Additionally, more biological assays, with different bone cell types (e.g., Saos-2, MC3T3) could be planned.

## Figures and Tables

**Table 1:** Content of aldehyde group determination results.

	m [g]	C_1_ [mol/dm^3^]	V_1_ [dm^3^]	C_2_ [mol/dm^3^]	V_2_ [dm^3^]	ALD	ALD [%]
sample 1	0.1008	0.2247	0.00890	0.2185	0.0075	0.573	57.3
sample 2	0.1021	0.2247	0.00895	0.2185	0.0075	0.583	58.3
sample 3	0.1020	0.2247	0.00890	0.2185	0.0075	0.566	56.6

m—mass of the sample [g]; C_1_—the concentration of NaOH solution [mol/dm^3^]; V_1_—the volume of NaOH solution [dm^3^]; C_2_—the concentration of HCl solution [mol/dm^3^]; V_2_—the volume of HCl solution [dm^3^]; ALD—aldehyde group content.

**Table 2:** The positions of characteristic bands for scaffolds crosslinked by dialdehyde starch and dialdehyde chitosan.

Sample		Amide A	C = N	Amide I	Amide II	C–N	Amide III	C–O
Coll/CTS/10SF	DAS	3326	1659	-	1552	1381	1241	1077
	DAC	3315	1658	1651	1550	1385	1241	1076
Coll/CTS/20SF	DAS	3326	1657	1652	1552	1381	1241	1077
	DAC	3296	1656	1648	1540	1386	1241	1076
Coll/CTS/30SF	DAS	3307	1657	1652	1549	1380	1241	1077
	DAC	3296	1656	1651	1540	1386	1241	1073
SF/Coll/10CTS	DAS	3304	1657	1650	1552	1387	1240	1080
	DAC	3307	1659	1650	1550	1386	1241	1077
SF/Coll/20CTS	DAS	3324	1659	-	1555	1380	1241	1077
	DAC	3399	1657	1650	1544	1380	1241	1076
SF/Coll/30CTS	DAS	3319	1658	1651	1552	1386	1240	1079
	DAC	3317	1658	1650	1543	1386	1240	1074
SF/CTS/10Coll	DAS	3298	1657	1652	1537	1379	1246	1070
	DAC	3305	1657	1648	1543	1386	1243	1076
SF/CTS/20Coll	DAS	3289	1655	1639	1532	1379	1245	1075
	DAC	3317	1659	1650	1543	1386	1241	1075
SF/CTS/30Coll	DAS	3304	1657	1652	1544	1381	1242	1076
	DAC	3307	1659	1651	1544	1386	1242	1077

**Table 3:** Porosity and density of biopolymeric scaffolds cross-linked with dialdehyde starch (DAS) and dialdehyde chitosan (DAC). Results are presented as mean ± standard error of the mean (*n* = 3).

Sample	Porosity [%]		Density [mg/cm^3^]	
	DAS	DAC	DAS	DAC
Coll/CTS/10SF	91.9 ± 4.5	93.7 ± 2.0	16.3 ± 2.0	15.2 ± 0.1
Coll/CTS/20SF	92.2 ± 1.4	91.0 ± 4.4	16.3 ± 0.2	14.8 ± 1.1
Coll/CTS/30SF	86.7 ± 1.0	86.0 ± 2.7	21.8 ± 2.4	14.6 ± 0.7
SF/Coll/10CTS	88.0 ± 3.8	94.2 ± 1.3	19.0 ± 2.1	16.5 ± 0.7
SF/Coll/20CTS	86.3 ± 3.4	92.6 ± 1.4	16.6 ± 1.9	12.3 ± 1.5
SF/Coll/30CTS	89.1 ± 3.3	90.0 ± 0.1	16.0 ± 1.5	11.1 ± 0.3
SF/CTS/10Coll	88.0 ± 0.4	88.0 ± 3.7	11.3 ± 1.1	15.3 ± 2.0
SF/CTS/20Coll	83.8 ± 1.0	87.8 ± 0.8	15.3 ± 0.2	17.4 ± 1.5
SF/CTS/30Coll	87.9 ± 0.1	87.9 ± 3.9	20.3 ± 0.2	27.5 ± 2.3

**Table 4:** The residue of scaffolds cross-linked with dialdehyde starch (DAS) and dialdehyde chitosan (DAC) after heating.

Sample	Residue [%] T = 600 °C	
	DAS	DAC
Coll/CTS/10SF	30.48	37.89
Coll/CTS/20SF	25.59	41.82
Coll/CTS/30SF	26.00	37.29
SF/Coll/10CTS	25.22	30.08
SF/Coll/20CTS	27.44	35.79
SF/Coll/30CTS	25.76	32.75
SF/CTS/10Coll	32.53	34.21
SF/CTS/20Coll	25.86	33.62
SF/CTS/30Coll	26.62	35.36

## Data Availability

The data presented in this study are available on request from the corresponding author.
